# Association between Gut Microbiota and Development of Gestational Diabetes Mellitus

**DOI:** 10.3390/microorganisms9081686

**Published:** 2021-08-08

**Authors:** Palin Sililas, Lingling Huang, Chanisa Thonusin, Suchaya Luewan, Nipon Chattipakorn, Siriporn Chattipakorn, Theera Tongsong

**Affiliations:** 1 Department of Obstetrics and Gynecology, Faculty of Medicine, Chiang Mai University, Chiang Mai 50200, Thailand; morin_mildmind@hotmail.com (P.S.); theera.t@cmu.ac.th (T.T.); 2 Neuroelectrophysiology Unit, Cardiac Electrophysiology Research and Training Center, Faculty of Medicine, Chiang Mai University, Chiang Mai 50200, Thailand; lingling_h@cmu.ac.th (L.H.); cthonusin@gmail.com (C.T.); nipon.chat@cmu.ac.th (N.C.); 3 Center of Excellence in Cardiac Electrophysiology Research and Training Center, Faculty of Medicine, Chiang Mai University, Chiang Mai 50200, Thailand

**Keywords:** Firmicutes/Bacteroidetes (F/B) ratio, gestational diabetes mellitus, microbiota, pregnancy

## Abstract

*Background:* It is well known that women with gestational diabetes mellitus (GDM) have gut dysbiosis. However, the dynamic alterations of gut microbiota in GDM are unclear. Additionally, the effects of maternal gut microbiota on the gut microbiota of their newborns remains controversial. The primary objective of this study is to determine the association between types and amounts of gut microbiota and development of gestational diabetes mellitus (GDM). *Methods*: Eighty-eight pregnant women, including 39 non-GDM and 49 GDM, and their 88 offspring were enrolled. Maternal feces were collected at the time of GDM diagnosis (24–28 weeks of gestation) and at before delivery (≥37 weeks of gestation). Meconium and the first feces of their newborns were also obtained. *Results:* from quantitative polymerase chain reaction (qPCR) showed that maternal Lactobacillales was decreased from baseline to the time before delivery in both non-GDM and GDM. Firmicutes/Bacteroidetes (F/B) ratio at before delivery was higher in the GDM group. However, there was no difference of neonatal gut microbiota between groups. *Conclusions:* Although we found only few gut microbiota that demonstrated the difference between GDM and non-GDM, gut microbiota may play a more important role in the development of severer GDM. Therefore, a further study comparing the gut microbiota composition among non-GDM, GDM with diet modification only, GDM with insulin therapy, GDM with successful treatment, and GDM with failure of treatment is needed.

## 1. Introduction

Gestational diabetes mellitus (GDM) is one of the most common complications of pregnancy, and the prevalence is varied from 1.8% to 22% [1]. According to American Diabetes Association (ADA) 2021 guideline of 2021, GDM is defined as glucose intolerance, which is first recognized during pregnancy. The diagnosis can be accomplished with either of two strategies: The one-step approach using 75-g oral glucose tolerance test (OGTT) or the two-step approach with a 50-g (non-fasting) screen followed by a 100-g OGTT for those who screen positive [2]. Pregnancy complicated with GDM is associated with an increased risk of obstetric and neonatal adverse outcomes such as preeclampsia, fetal distress, fetal macrosomia, operative delivery, postpartum infection, and neonatal hypoglycemia. Additionally, the offspring of diabetic mothers have a higher risk of developing diabetes mellitus, hypertension and dyslipidemia, known as metabolic syndrome and cardiovascular diseases later in their lives. Therefore, understanding GDM pathogenesis identification of risk factors and providing appropriate prevention and treatment are of great importance.

The mucosal surface and lumen of the respiratory tract, gastrointestinal (GI) tract, urinary tract, and reproductive tract are composed of microbiota communities, known as “microbiota” [3,4,5]. The GI tract, especially the colon, is populated with the largest microbiota density, defined as “gut microbiota”. Gut microbiota supports food digestion, short-chain fatty acid production, vitamin synthesis, and mucosal barrier function. Importantly, interactions between host cells and gut microbiota result in shaping host metabolism and immune response [6,7]. An imbalance of gut microbiota population, such as an increase in harmful bacteria and a decrease in beneficial bacteria, is associated with several underlying diseases such as metabolic syndrome, allergic diseases, some types of cancer, and neurological diseases [8,9,10]. Previous studies showed that gut dysbiosis plays a vital role in many pregnancy complications, including preeclampsia, prematurity, and metabolic dysfunction [11,12,13]. However, the role of gut dysbiosis in GDM remains unclear. It was reported that gut dysbiosis in GDM women was mainly characterized by changes in microbiota diversity, including alpha-diversity, a diversity of species within the same individual, and beta-diversity: An inter-individual species diversity. Moreover, changes in different taxa including phylum, genus, and species levels were also exhibited in GDM, especially in mid and late gestation [14,15]. At the phylum level, an increase in Firmicutes and a decrease in Bacteroidetes, as well as increased Firmicutes/Bacteroidetes (F/B) ratio in late pregnancy were found in the GDM group. Previous studies indicated that a higher F/B ratio was found in obesity and metabolic syndrome, also positively associated with low-grade inflammation and energy storage [16]. Accordingly, gut dysbiosis may theoretically interfere with glucose metabolism and plays a role in pathogenesis of GDM. To date, studies on the association between gut microbiota and GDM have been rarely reported.

However, the information regarding dynamic changes in maternal gut microbiota composition of GDM, when compared with those of the non-GDM cases, is limited. Moreover, the effects of maternal gut microbiota composition, both with and without GDM, on the gut microbiota composition of their offspring remains to be understood. Therefore, we aimed to compare the alterations of maternal gut microbiota composition from the second trimester at baseline (at the time of GDM diagnosis) to the third trimester at before delivery in GDM versus the non-GDM individuals. Additionally, the gut microbiota composition was compared between the offspring of GDM and those of non-GDM, in both meconium and feces.

## 2. Materials and Methods

### 2.1. Study Protocol

Singleton pregnant women who attended antenatal care at Maharaj Nakorn Chiang Mai Hospital, Chiang Mai University, Chiang Mai, Thailand were enrolled in this study. The enrollment period was August 2019 to February 2020, and the informed consent process was performed for all participants. The study was conducted under the ethical approval of the institutional review boards (Study Code: OBG-2562-06451(Approval date: 8 August 2019)). The inclusion criteria were 1) singleton pregnancy with gestational age between 24 and 28 weeks, based on the reliable last menstrual period or the ultrasound in the first half of pregnancy. The exclusion criteria were (1) having any underlying medical diseases prior to pregnancy, (2) multifetal gestation, (3) fetal or neonatal anomaly, (4) receiving any medication or supplement that could affect the glucose level such as corticosteroids, beta-blockers, and antibiotics at the time of GDM diagnosis and (5) incomplete data or unknown pregnancy outcomes. The participants were categorized into 2 groups, including (1) GDM and (2) non-GDM group. GDM was diagnosed in accordance with the National Diabetes Data Group (NDDG) criteria, in which 100-g oral OGTT was done after a glucose challenge test >140 mg/dL. Pregnant women who exhibited two or more blood glucose levels higher than the cut-off values (fasting—105, 1 h—190, 2 h—165, and 3 h—145 mg/dL) were classified as GDM. Importantly, women with abnormal OGTT according to the Carpenter and Coustan criteria [17] were not included in the non-GDM group. Baseline characteristics, as well as pregnancy and neonatal outcomes, were also prospectively collected.

### 2.2. Sample Size Calculation

To investigate the alterations of gut microbiota in GDM mothers and their newborns, the fasting blood glucose is a key indicator for the estimation of sample size. The sample size of the study was calculated by the GPower 3.1 software according to the Student’s *t*-test for comparison the independent means between two groups. The calculation of effect size was performed using the formula below: Effect size = (Mean 1 − Mean 2)/SD1.Data for the calculation of effect size were obtained from fasting blood glucose values published by a previous study, which were 4.7 ± 0.5 mmol/L for GDM and versus 4.3 ± 0.3 mmol/L for non-GDM with *p*-value < 0.001 [14]. Therefore, effect size = (4.7 − 4.3)/0.5 = 0.8; α = 0.05 (represent the expected significance); 1 β = 0.95 (represent expected validity of inspection). The result from GPower 3.1 software showed that the estimated sample size was 35.

### 2.3. Sample Collection

Maternal blood and stool were collected at the time GDM diagnosis (24–28 weeks of gestation) and at before delivery (≥37 weeks of gestation). For the blood, 5 mL of venous blood was obtained after an overnight fasting for the measurement of glycated hemoglobin (HbA1c), lipid profile (total cholesterol, triglyceride, and low-density lipoprotein; LDL). Fasting glucose and 2-h postprandial glucose were also measured from the capillary blood. A stool sample was self-collected by the participants in the morning on the same day as the blood test. In details, 5 g of stool were collected in a sterile tube under antiseptic handling. The samples were then transferred to the lab and stored at −80 °C for DNA extraction. Additionally, neonatal meconium and first feces of newborns were collected by medical staffs using the same technique as maternal stool collection. The meconium and first feces were obtained within 24 and 48 h after delivery, respectively.

### 2.4. Gut Microbiota Analysis

Bacterial genomic DNA were extracted from human feces using a commercial genomic DNA isolation kit (QIAGEN, Hilden, Germany). Briefly, feces (0.25 g) was homogenized in QIAGEN ASL lysis buffer by a Minibeadbeater (BioSpec Products, Bartlesville, OK, USA) and following the manufacturer’s instruction to extract the bacterial genomic DNA in human feces. Fractions of bacterial microbiota population (F/B ratio, *Enterobacteriaceae* and *Bifidobacteria*) were quantified using real-time quantitative polymerase chain reaction (qPCR) as described previously. Extracted bacterial genomic DNA were diluted 1:10 and used 0.04 mL as the template for SYBR-green based real-time PCR using the primers listed in Table 1. The gene copy numbers of each bacterial population were determined based on standard curves generated from fragments of bacterial 16S rRNA genes; *Eubacteria: Ruminococcus. productus* (ATCC 27340D), *Clostridiales: R. productus* (ATCC 27340D), *Lactobacillales/Bacillales*: *L. acidophilus (ATCC 4357D), Bacteroidetes/Actinobacteria: B. fragilis (ATCC 25285D), Actinobacteria/Bifidobacterium: Bifidobacterium adolescentis* (ATCC 15703) and *Enterobacteriaceae*: *E. coli* K-12 (TOP10) cloned into pCR2.1 (TOPO TA cloning kit, Invitrogen, Carlsbad, CA, USA)) as templates [18]. The percentage of Firmicutes was calculated from qPCR using standard curves generated by pSW192 and pSW193. The percentage of each bacterial phylum was determined by dividing with Eubacteria as previously described [18]. Viable counts of Enterobacteriaceae in human feces were determined by a serial dilution method with a selective MacConkey agar (HiMedia, Mumbai, India) at 37 °C for 16–18 h.

### 2.5. Statistical Analysis

Statistical analyses were performed using SPSS version 21.0 (IBM Corp. Released 2012; IBM SPSS Statistics for Windows, Armonk, NY, USA), and GraphPad Prism 8 (San Diego, CA, USA). For categorical variables, Fisher’s exact test was used for the comparison between groups. The continuous variables were also compared between groups and timepoints using either Student’s *t*-test (for metabolic parameters) or Mann–Whitney U test (for gut microbiota). These variables were reported as mean ± standard deviation (SD) or mean ± standard error of mean (SEM). The *p*-value less than 0.05 was considered as statistical significance.

## 3. Results

### 3.1. Clinical Characteristics and Metabolic Parameters

Clinical characteristics and metabolic parameters are detailed in Table 2. A total of 88 pregnant women were recruited with complete follow-up, including 39 cases in the non-GDM group and 49 cases in the GDM group. At the time of GDM diagnosis, maternal age, pre-pregnant body mass index (BMI), gestational age, and lipid profiles were not different between groups. However, fasting blood glucose, 2-h postprandial glucose, and HbA1c levels were higher in the GDM group. At before delivery, fasting blood glucose, 2-h postprandial glucose, and HbA1c levels remained higher in the GDM women, whereas lipid profiles remained no different between groups. The gestational age at delivery were significantly lower in the GDM group (38.3 ± 1.0 weeks) than that of the non-GDM controls (39.0 ± 0.9 weeks). Nevertheless, the delivery of all participants was term delivery.

Eighty-eight newborns from all 88 pregnant women (39 for non-GDM and 49 for GDM) were also included in this study. The percentage of cesarean section, birth weight, and the percentage of large for date were not different between the two groups.

### 3.2. Gut Microbiota

**Figure 1:**
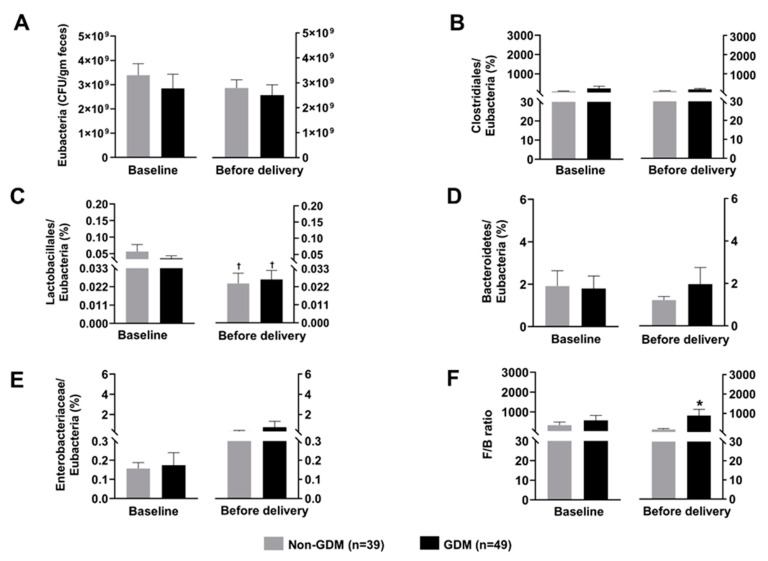
Composition of maternal gut microbiota at baseline (time of GDM diagnosis) and before delivery; (**A**): Total count of *Eubacteria*, (**B**): Normalized abundance of *Clostridiales*, (**C**): Normalized abundance of *Lactobacillales*, (**D**): Normalized abundance of *Bacteroidetes*, (**E**): Normalized abundance of *Enterobacteriaceae*, (**F**): F/B ratio. Data are reported as mean ± SEM. The Mann–Whitney U test was used to determine the difference between groups. * *p* < 0.05 vs. non-GDM group, † *p* < 0.05 vs. the same group at the baseline GDM: Gestational diabetes mellitus; F/B ratio: *Firmicutes/Bacteroidetes* ratio.

The gut microbiota composition of the mothers is shown in Figure 1. At the time of GDM diagnosis, the abundance of *Eubacteria* was not different between groups (Figure 1A). After normalization by the abundance of *Eubacteria*, the abundance of *Clostridiales, Lactobacillales, Bacteroidetes, Enterobacteriaceae*, and F/B ratio were not different between groups as well (Figure 1B–F). At before delivery, F/B ratio was higher in the GDM subjects than that of the non-GDM controls (Figure 1F). Additionally, the abundance of *Lactobacillales* was significantly decreased from baseline (at the time of GDM diagnosis) to the time before delivery in both non-GDM and GDM groups (Figure 1C).

**Figure 2:**
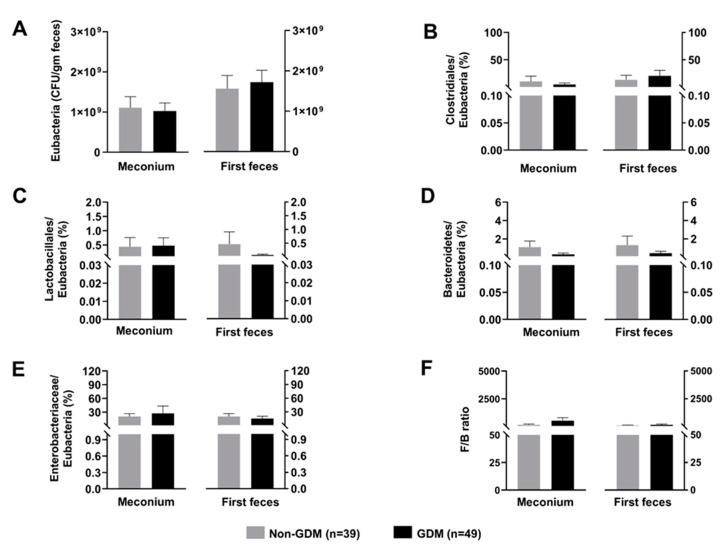
Composition of neonatal gut microbiota; (**A**): Total count of *Eubacteria*, (**B**): Normalized abundance of *Clostridiales*, (**C**): Normalized abundance of *Lactobacillales*, (**D**): Normalized abundance of *Bacteroidetes,* (**E**): Normalized abundance of *Enterobacteriaceae*, (**F**): F/B ratio. Data are reported as mean ± SEM. The Mann–Whitney U test was used to determine the difference between groups. GDM: Gestational diabetes mellitus; F/B ratio: *Firmicutes/Bacteroidetes* ratio.

The gut microbiota composition of the newborns is shown in Figure 2. None of the gut microbiota showed the significant different between groups, both in meconium and the first feces. Moreover, the alterations of gut microbiota composition from meconium to the first feces were not observed.

## 4. Discussion

We observed a reduction in *Lactobacillales* from baseline (at the time of GDM diagnosis) to the time before delivery (≥37 weeks gestation). Additionally, F/B ratio was found higher in GDM mother, when compared to their non-GDM counterparts, at the time before delivery. However, these alterations were not observed in meconium and the first feces of their newborn. The main insight gained from this study is that amounts of most gut microbiome in pregnancies with GDM are not significantly different from those in non-GDM, different from previous studies which suggest that microbiome dysbiosis might play a major role in the development of GDM. However, at the baseline period (24–28 weeks of gestation) the GDM group tended to have a decrease in *Lactobacillus* amounts, favoring insulin resistance. Thus it is possible that, at 24–28 weeks, GDM is still in very early phase of metabolic changes and the difference in microbiota might be subtle. If the difference exists, it seems to be more obvious in late gestation (>37 weeks). However, in this study, even in late gestation, the amounts of gut microbiota were also not significantly different in most types. Nevertheless, we provided intervention to control blood glucose, either by diet control or insulin in cases of GDM. Such intervention might obliterate the natural course of the effects secondary to gut microbiota.

With advancing gestational age, the types and amounts of gut microbiota were significantly changed, when compared to those in baseline periods. Such changes were associated with an increase in insulin resistance, promoting GDM, for examples a significant decrease in the number of *Lactobacillus*, etc. However, surprisingly, in GDM group the changes were not so obvious when compared to those in the non-GDM group. This was likely caused by the therapeutic intervention as mentioned above. It is well-established that, with advancing gestational age, insulin resistance is increased as a normal physiologic change, mainly caused by human placental lactogen (HPL). Thus the changes with gestational age found in this study were unlikely caused by gut microbiota. It is possible that gut microbiota have only minor effect on increased insulin resistance but the large size effect of HPL might obscure the minor effect of gut microbiota. To show the significant effect of microbiota on insulin resistance and development of GDM may need a much greater sample size. However, based on this study, though gut microbiota can modify risk of insulin resistance, it may have only little clinical impact on GDM.

In our study, we used the criteria of NDDG for the diagnosis of GDM. Even though ADA recommends the diagnostic thresholds of Carpenter-Coustan, which is lower than NDDG [2], the American College of Obstetricians and Gynecologists (ACOG) recommends either of two sets of diagnostic thresholds for the 100-g OGTT—Carpenter-Coustan or NDDG [19]. A prior study also showed that the outcomes of GDM diagnosed using Carpenter-Coustan did not differ from that of NDDG criteria [17]. Importantly, women with abnormal OGTT according to the Carpenter and Coustan criteria were not included in our non-GDM group in order to avoid the conflict results.

*Lactobacillales* was found to have a probiotic property, in which it could ameliorate insulin resistance-induced gut dysbiosis and inflammation [20]. According to our knowledge, a decrease in *Lactobacillales* from the second to third trimester has never been reported. For this reason, molecular mechanisms that mediate a reduction in the probiotics from mid-to-late gestation should be further identified. Nevertheless, our finding was consistent with several prior studies demonstrating the benefits of probiotics supplement in pregnant mothers [21,22]. These benefits include the improvement of insulin sensitivity, decreased systemic inflammation, reduced the risk of preeclampsia, and decreased the incidence of preterm delivery [23,24,25,26,27,28,29,30].

The F/B ratio is considered to be a marker of low-grade systemic inflammation in obesity and insulin resistance [31]. Consistent with another previous study [16], our result showed that F/B ratio at the third trimester was higher in GDM patients, when compared with the non-GDM controls. Interestingly, F/B ratio is the only gut microbiota that revealed the significant difference between non-GDM and GDM in our study. In other words, F/B ratio is a more sensitive marker of GDM than *Eubacteria, Clostridiales, Lactobacillales, Bacteroidetes,* and *Enterobacteriaceae*.

In contrast with some prior studies discovering the huge difference of gut microbiota composition between non-GDM and GDM mothers [14,15,32], our study demonstrated only minor changes in gut microbiota composition between these two groups. However, this was consistent with a recent study, in which the minor changes in gut microbiota composition were also observed in GDM women, when compared to the non-GDM controls [33]. These minor alterations may be due to too less severity of our GDM cases. Hence, a further study investigating the maternal gut microbiota composition in the different degree of severity of GDM should be established.

Regarding the gut microbiota composition of the offspring, we did not find any difference of gut microbiota between the newborns of non-GDM and GDM mothers. This was inconsistent with some previous studies, in which they revealed gut dysbiosis in the offspring of GDM cases [34,35,36]. Similar to the finding in their mothers, this negative finding may be explained by too less severity of our GDM participants. Therefore, a future study determining the neonatal gut microbiota composition in the different degree of severity of GDM should be conducted.

Interestingly, the prevalence of GDM was relatively high in this study (approximately 50% among women at risk), when compared to that reported in Western studies. During the same period, the prevalence of GDM in our general obstetric population was 27.2%. This is consistent with what is already known that Southeast Asian population has higher prevalence of GDM. Perhaps, diagnostic criteria of GDM should be ethnicity-specific. The current criteria which is commonly used is too sensitive for our population. It is possible that many cases of GDM in this study (Thai population) was misclassified. If this is true, many cases of non-GDM were allocated into the GDM group and this might obscure the difference of gut microbiota types and amount between both groups, if it existed.

The strengths of this study are as follows: (1) Longitudinal prospective collections, more reliable in determination of gut microbiota change from the baseline period to late gestation period; (2) collections of specimens including both maternal and neonatal gut microbiota; (3) reliable advanced technique in microbiome DNA isolation. The weaknesses of this study include: (1) Our study did not study gut microbiome in the cases of pregestational DM, initially excluded. If gut microbiota is associated with DM, it could show more obvious dysbiosis than seen in GDM. (2) The sample size was relatively small for other pregnancy outcomes, though adequate to address the primary objective. Additionally, we did not use 16S sequencing or metagenomic sequencing for the gut microbiota analysis, and therefore the gut microbiota profiles were reported only at levels above genus. This also limited us to identify the difference of alpha- and beta-diversity between GDM and non-GDM. Importantly, we did not subcategorize our GDM participants into the different severity, which were “only diet modification needed”, “insulin needed”, “successful treatment”, and “failure of treatment”. This may be a reason why we discovered only few gut microbiota that displayed the difference between GDM and non-GDM, both in the mothers and their newborns.

## 5. Conclusions

Even though we observed that there were only few gut microbiota that exhibited the difference between GDM and non-GDM, gut microbiota may play a more crucial role in the development of severer GDM. Hence, a future study comparing the gut microbiota composition among non-GDM, GDM with diet modification only, GDM with insulin therapy, GDM with successful treatment, and GDM with failure of treatment should be established.

## Figures and Tables

**Table 1:** Primers using in real-time qPCR [19].

Gut Microbiota	Target Gene	Sequence
*Eubacteria*	16S rRNA	5′-ACTCCTACGGGAGGCAGCAGT-3′ 5′-ATTACCGCGGCTGCTGGC-3′
*Firmicutes/Clostridiales*	16S rRNA	5′-ACTCCTACGGGAGGCAGC-3′ 5′-GCTTCTTTAGTCAGGTACCGTCAT-3′
*Firmicutes/Lactobacillales*	16S rRNA	5′-AGCAGTAGGGAATCTTCCA-3′ 5′-CACCGCTACACATGGAG-3′
*Bacteroidetes*	16S rRNA	5′-GGTTCTGAGAGGAGGTCCC-3′ 5′-GCTGCCTCCCGTAGGAGT-3′
*Proteobacteria/Enterobacteriaceae*	16S rRNA	5′-GTGCCAGCMGCCGCGGTAA-3′ 5′-GCCTCAAGGGCACAACCTCCAAG-3′

**Table 2:** Clinical characteristics and metabolic parameters.

Data	Non-GDM (*n* = 39)	GDM (*n* = 49)	*p*-Value
Maternal characteristics			
Age (years)	30.9 ± 5.5	32.6 ± 4.6	0.124
Pre-pregnant BMI (kg/m^2^)	22.9 ± 4.5	24.5 ± 5.0	0.132
Baseline (GA 24–28 weeks)			
GA at sample collection (weeks)	24.8 ± 0.9	25.0 ± 1.1	0.375
FBG (mg/dL)	78.7 ± 8.8	95.1 ± 11.2	<0.001
2-h PP (mg/dL)	110.0 ± 18.4	137.3 ± 22.9	<0.001
HbA1c (%)	4.8 ± 0.4	5.0 ± 0.3	0.017
Total cholesterol (mg/dL)	236.3 ± 35.5	237.2 ± 39.9	0.917
Triglyceride (mg/dL)	208.6 ± 80.5	214.3 ± 89.2	0.754
LDL (mg/dL)	152.0 ± 29.9	151.6 ± 42.6	0.956
Before delivery (GA ≥ 37weeks)			
GA at delivery (weeks)	39.0 ± 0.9	38.3 ± 1.0	0.003
FBG (mg/dL)	76.7 ± 8.4	89.4 ± 12.6	<0.001
2-h PP (mg/dL)	109.8 ± 14.8	122.0 ± 14.8	<0.001
HbA1c (%)	4.9 ± 0.4	5.1 ± 0.4	0.005
Total cholesterol (mg/dL)	232.6 ± 34.1	235.0 ± 39.2	0.764
Triglyceride (mg/dL)	234.3 ± 91.5	245.9 ± 81.7	0.511
LDL (mg/dL)	160.6 ± 40.8	156.9 ± 37.3	0.658
Newborn characteristics			
Cesarean section (case, %)	9 (23.1%)	18 (36.7%)	0.168
Birth weight (gm.)	3087 ± 321.3	3079 ± 369.9	0.909
Large for date (case, %)	2 (5.13%)	5 (10.2%)	0.456

Data are reported as mean ± SD. Either Fisher’s exact test or Student’s *t*-test was used for the comparison between groups. GDM: Gestational diabetes mellitus; BMI: Body mass index; GA: Gestational age; FBG: Fasting blood glucose; 2-h PP: 2-h postprandial glucose; HbA1C: Glycated hemoglobin; LDL: Low-density lipoprotein.

## Data Availability

The datasets analyzed during the current study are available from the corresponding author upon reasonable request.
